# Effect of nutrient supplementation on somatic growth in very low birth weight infants: a protocol for a systematic review and network meta-analysis

**DOI:** 10.1186/s13643-025-03027-3

**Published:** 2026-02-19

**Authors:** Muneerah Satardien, Michael Mccaul, Evette Van Niekerk, Mirjam van Weissenbruch, Lizelle Van Wyk

**Affiliations:** 1 Department of Paediatrics and Child Health, Faculty of Medicine and Health Sciences, Stellenbosch University, Cape Town, South Africa; 2 Division of Epidemiology and Biostatistics, Stellenbosch University, Cape Town, South Africa; 3 Department of Dietetics, Faculty of Medicine and Health Sciences, Stellenbosch University, Cape Town, South Africa; 4 Emma Children’s Hospital, Division IC Neonatology, VU University, Amsterdam, The Netherlands

**Keywords:** Very low birth weight infants, Nutrient supplementation, Network meta-analysis, Somatic growth, Extrauterine growth restriction

## Abstract

**Aim:**

No consensus exists on the ideal nutrition for preterm infants. This leads to significant practice variation. Fortification of expressed breast milk (EBM) and nutrient supplementation of the preterm infant’s diet have become common practices to enhance growth. This systematic review aims to review the comparative effectiveness of oral macro- and micronutrient supplements in enhancing post-natal growth for preterm infants. Additionally, to identify critical gaps in the current recommendations for nutrient supplements.

**Methods:**

We will conduct a systematic review and network meta-analysis (NMA). Websites and databases will be searched for randomised controlled trials and prospective observational studies investigating oral macro and micro-nutrient supplementation to improve in-hospital somatic growth for premature very low birth weight infants (VLBWIs, < 1500 g). Two review authors will assess full-text English publications between 2010–2024 for potentially relevant studies for inclusion, independently and in duplicate, utilising an eligibility form based on the inclusion criteria. The selection process will be demonstrated graphically utilising a PRISMA flow diagram. Details and characteristics of excluded studies will be provided.

A network meta-analysis (NMA) will be done using a frequentist approach and multivariate meta‐analysis. Random-effect models will be employed to estimate all relative treatment effects. Stata will be used for data analysis. All possible comparisons, containing the effect size and 95% CIs, will be reported in tabular form. If the assumptions that preserve the validity of the NMA are not met, a pairwise meta-analysis will be done. If the criteria for pair-wise meta-analysis are not met, only direct comparisons will be made, and a narrative description of the findings will be presented employing the synthesis without meta-analysis guidelines.

The GRADE approach will be used to review the certainty of the evidence. For each comparison, the overall certainty of evidence for the primary outcomes will be evaluated. Estimates of the direct and/or indirect evidence of the NMA will be provided.

**Results:**

The NMA will generate comparative rankings of nutrient supplementation interventions and evaluate their effectiveness in improving in-hospital growth. Results will be presented as summary tables and SUCRA rankings.

**Conclusion:**

This NMA will provide evidence-based guidance on optimal nutrient supplementation strategies to enhance postnatal growth in VLBWIs, addressing a critical knowledge gap in neonatal nutrition practices.

**Systematic review registration:**

This protocol has been registered with PROSPERO.

Registration number: CRD420250650341.

**Supplementary Information:**

The online version contains supplementary material available at 10.1186/s13643-025-03027-3.

## Introduction

Preterm very low birth weight infants (VLBWIs) are at high risk of postnatal growth failure due to limited nutrient reserves, physiological immaturity, and frequent co-morbidities [1–3]. Adequate provision of macro- and micronutrients during the early postnatal period is essential to support somatic growth and neurodevelopment [4, 5].

Despite attempts to optimise early nutrition for VLBWIs, extrauterine growth restriction (EUGR) remains common globally, with incidences ranging from 13 to 80%, depending on the definition used and clinical context [6, 7].

To support optimal growth, a wide range of nutrient supplementation strategies are used in neonatal units, including protein, lipid, mineral, and multi-nutrient formulations, as well as fortified expressed breast milk [8–14]. Human milk fortifiers (HMFs) and nutrient supplements have been shown to improve weight growth velocity (WGV) [10, 11, 15]. Appendix 3 depicts the various available fortifiers and oral supplements used, and their impact on post-natal in-hospital growth.

### What we already know

Multiple evidence syntheses in the form of systematic reviews already exist related to nutrient supplements for preterm infants, with clear evidence on the beneficial role of these nutrients on in-hospital growth, yet findings remain fragmented [11–13, 16–19]. All of these syntheses, however, review the effect of individual nutrients or individual fortifiers in isolation, without offering a comparative overview of how different supplements perform relative to each other.

### Importance of the review

Given these limitations in the existing evidence base, a comprehensive synthesis is still lacking, as no synthesis currently identifies which specific oral nutrient interventions, or combinations thereof, provide the greatest benefit for in-hospital postnatal growth in VLBWIs. This limits clinicians’ ability to select the most effective supplementation strategy within diverse care settings.

A network meta-analysis (NMA) enables the synthesis of both direct and indirect comparisons across diverse nutrient interventions, integrating all available evidence to determine which interventions offer the greatest benefit. This provides clearer guidance than traditional approaches, which assess interventions in isolation.

This NMA will provide comparative effectiveness rankings of enteral nutrient supplementation strategies for VLBWIs, offering evidence-based guidance to inform clinical nutritional practices.

## Objectives

This review will evaluate the effectiveness of oral nutrient supplements in improving in-hospital post-natal growth in very low birth weight infants (VLBWIs).

It will assess the effects of oral nutrient supplementation of macronutrients and micronutrients (i.e., macro-minerals, trace elements, and vitamins) on in-hospital post-natal growth in premature VLBWIs (Table 1).

**Table 1:** Recommended ESPGHAN daily requirements for preterm infants

	**ESPGHAN 2022 recommendations **[15]
Fluid, ml/kg/day	150–180 (135–200)
Energy, kcal/kg/day	115–140 (160)
Protein, g/kg/day	3.5–4.0 (−4.5)
Fat, g/kg/day	4.8–8.1
Linoleic acid, mg/kg/day	385–1540
α-Linolenic acid, mg/kg/day	≥ 55
DHA, mg/kg/day	30–65
ARA, mg/kg/day	30–100
EPA, mg/kg/day	< 20
Carbohydrate, g/kg/day	11–15 (−17)
Sodium, mmol/kg/day	3.5–5.0 (8.0)
Chloride, mmol/kg/day	3.5–5.0 (8.0)
Calcium, mmol/kg/day	3.0–5.0
Potassium, mmol/kg/day	2.3–4.6
Phosphorus, mmol/kg/day	2.2–3.7
Magnesium, mmol/kg/day	0.4–0.5
Iron, mg/kg/day	2.0–3.0 (−6.0)
Zinc, mg/kg/day	2.0–3.0
Copper, µg/kg/day	120–230
Selenium, µg/kg/day	7–10
Manganese, µg/kg/day	1–15
Iodine, µg/kg/day	11–55
Chromium, µg/kg/day	0.03–2.25
Molybdenum, µg/kg/day	0.3–5.0
Thiamine (B1), µg/kg/day	140–290
Pantothenic acid, mg/kg/day	0.6–2.2
Biotin, µg/kg/day	3.5–15
Niacin, µg/kg/day	1100–5700
Ascorbic acid (vit C), mg/kg/day	17–43
Riboflavin (B2), µg/kg/day	200–430
Pyridoxine, µg/kg/day	70–290
Folic acid, µg/kg/day	23–100
Cobalamin (B12), µg/kg/day	0.1–0.6
Vitamin A, IU/kg/day	1333–3300 (400–1000 µg retinol ester/kg/day)
Vitamin D, IU/kg/day	400–700 (< 1000)
Vitamin E, mg/kg/day	2.2–11
Vitamin K, µg/kg/day	4.4–28

## Methodology

The review will be reported using the PRISMA (Preferred Reporting Items for Systematic Reviews and Meta‐Analyses) 2020 checklist. This study protocol was registered with PROSPERO (registration number: CRD420250650341).

The review will include studies enrolling very low birth weight infants (VLBWIs), defined as infants with a birth weight below 1500 g. Eligible studies will evaluate nutrient supplementation interventions, which may include macronutrients (protein, lipids) or micronutrients (vitamins, macro-minerals, or trace elements), either as single or multi-nutrient formulations. The comparators will include other supplementation regimens, different dosages or concentrations, or unfortified human milk serving as the control. The primary outcomes of interest are indicators of somatic growth-specifically weight, length, and head circumference velocity or Z-scores, measured during hospitalisation or at discharge. We will include randomised controlled trials (RCTs) and prospective observational studies published between 2010 and 2024.

### Criteria for considering studies for this review

#### Types of studies

Randomised controlled trials (RCTs) or prospective observational (PO) trials will be included. Retrospective observational, non-experimental, and non-randomised designs will be excluded. Only data from RCTs in full‐text English publications from 2010–2024 will be included.

#### Types of participants

Studies should include hospitalised VLBW preterm infants receiving fortification and/or other macro or micronutrient supplementation for growth purposes.

#### Types of interventions

Studies should include one or more of the following interventions aimed at improving in-hospital post-natal growth.

#### Formulation of the intervention

- Protein supplementation (single nutrient)
- Lipid supplementation (single nutrient)
- Single micronutrient supplementation (e.g., zinc, iron, selenium, vitamins, sodium)
- Multi-nutrient fortifier + additional single nutrient
- Unfortified human milk or standard feeds (comparator)

Only enteral supplementation strategies will be considered for network analysis.

#### Interventions: nodes of the NMA

The interventions evaluated in this review include enteral nutrition strategies incorporating nutrient supplementation aimed at enhancing in-hospital postnatal growth among very low-birth-weight infants. Each intervention node within the planned NMA represents a distinct formulation or combination of macro- or micronutrients (protein, lipids, minerals, trace elements, or vitamins) administered either alone or in fortified human milk.

To minimise clinical heterogeneity, the intervention nodes include only enteral nutrient supplements. Parenteral nutrition will not be included as an intervention node; however, its use will be recorded and described as a co-intervention, given its potential role as a confounder in influencing early postnatal growth outcomes.

### Comparators

**Fig. 1:**
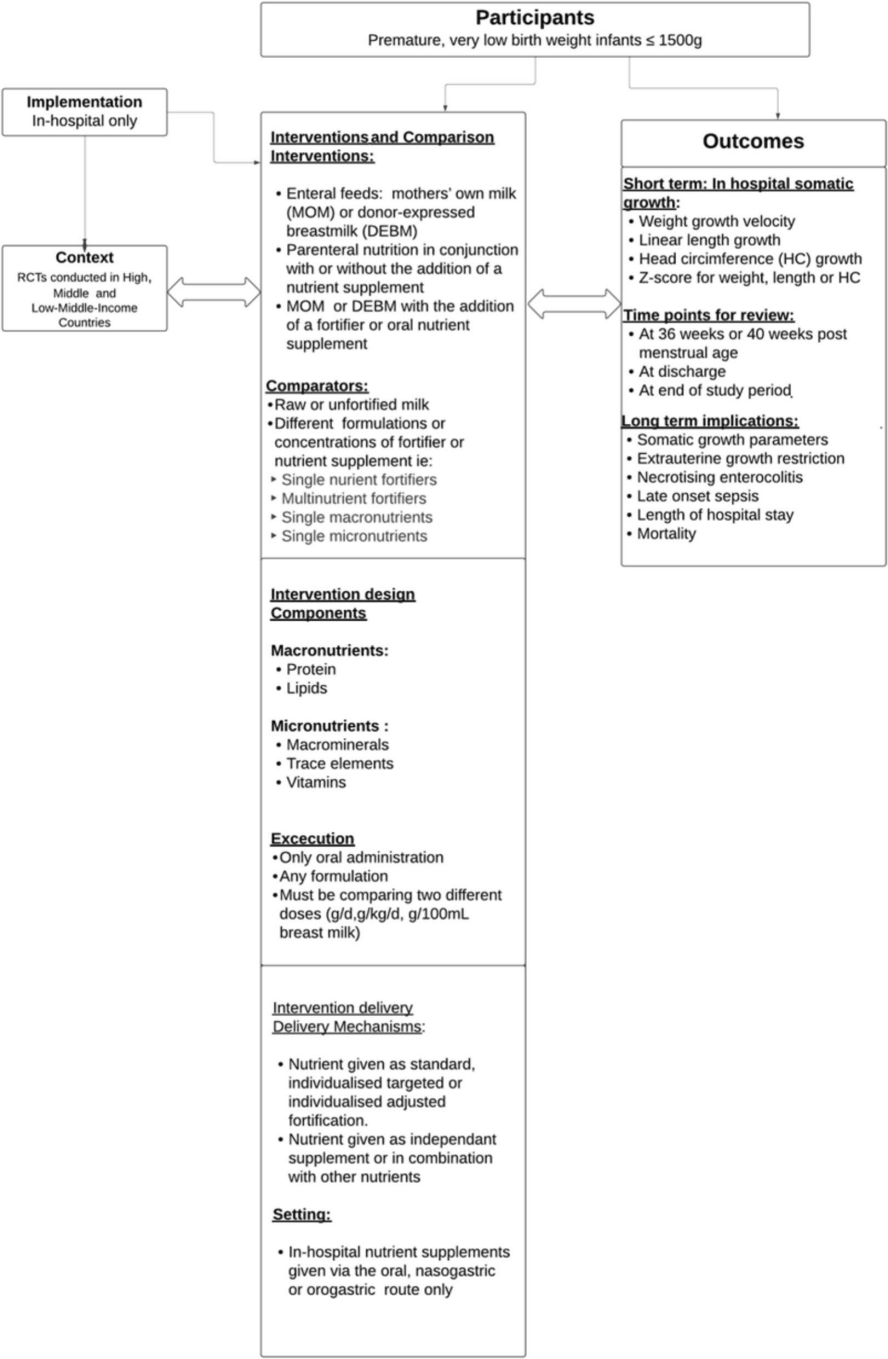
Study system-based logic model

Comparators will include alternative supplementation regimens, differing dosages or concentrations, or unfortified expressed breast milk serving as standard care. The conceptual network linking participants, interventions, and outcomes is illustrated in Fig. 1.

### Types of outcome measures

#### Primary outcomes

The primary outcomes of interest will include (1) indicators of somatic growth and (2) length of hospital stay, which will be recorded as an outcome reflecting recovery and growth trajectory.

Growth outcomes will encompass weight growth velocity (measured in grams per kilogram per day, grams per day, or grams per week), which will be evaluated at 36- or 40-weeks postmenstrual age (PMA), at hospital discharge, or at the end of the study period.

#### Secondary outcomes

The secondary outcomes will focus on absolute weight, linear length gain (centimetres per week), and head circumference (HC) growth (centimetres per week). Corresponding Z-scores for weight, length, and head circumference at the same time points described above will also be analysed.

Secondary outcomes will also include clinically significant morbidities associated with nutritional status, including necrotizing enterocolitis (NEC), late-onset sepsis (LOS), and mortality (see Appendix for detailed outcome definitions). The incidence of extra-uterine growth restriction (EUGR) will be assessed using any reported decline in weight Z-score or percentile from birth to discharge or study end.

### Eligibility criteria

#### Inclusion of studies

This review will include randomized controlled trials (RCTs) and prospective observational (OP) studies conducted in neonatal intensive care units (NICUs) or special care baby units between 2010 and 2024. The date limit reflects advances in neonatology and the evolving understanding of the nutritional requirements of very low birth weight infants (VLBWIs) over the past two decades. This restriction ensures that included studies are methodologically comparable and relevant to present-day clinical practice since significant shifts in nutrient recommendations occurred following the 2010 and 2022 ESPGHAN guidelines [15, 20]. Eligible studies will involve preterm infants with a gestational age of ≤ 35 weeks and/or a birth weight below 1500 g, who received fortification of enteral feeds and/or oral macro- or micronutrient supplementation aimed at improving postnatal in-hospital growth.

In addition to published studies, grey literature such as conference abstracts, dissertations, and theses (for example, those indexed in *ProQuest Dissertations & Theses*) will be considered to reduce publication bias.

#### Exclusion of studies

Studies will be excluded if they are not written in the English language or if they focus on unrelated forms of supplementation, such as prebiotics, probiotics, synbiotics, or human milk oligosaccharides (HMOs), since these do not represent direct macronutrient or micronutrient interventions relevant to the present review.

A conceptual framework to depict the interaction between the participants and interventions is shown in Fig. 1.

### Search methods for identification of studies

Studies that meet the inclusion criteria and are published in the English language only will be considered.

#### Identification of studies

To identify all relevant studies, a comprehensive literature search will be performed in consultation with an information specialist and neonatal-nutrition experts, in addition to an analysis of terms used in studies identified in exploratory searches. Each search strategy will be peer-reviewed using the PRESS checklist (Peer Review of Electronic Search Strategies) to ensure accuracy and completeness. The strategy will integrate both controlled vocabulary and free-text terms relating to population, intervention, and study context, combined with validated filters for randomized controlled and prospective observational designs. Search strategies will be adapted for each database using appropriate syntax and controlled vocabulary.

### Information sources

#### Electronic searches

Searches will be conducted across the following databases: Cochrane CENTRAL, PubMed, CINAHL (EBSCOHost), Scopus, Web of Science (Social Sciences Citation Index and Science Citation Index Expanded), Conference Proceedings Citation Index, and the WHO International Clinical Trials Registry Platform.

Each database strategy will be tailored to its syntax and reported in the final review, with a sample PubMed strategy provided in Appendix 2.

#### Additional resources

To ensure comprehensive coverage, additional searches will include screening the reference lists of eligible reviews and studies, conducting forward citation chasing using the Citation Chaser tool (https://estech.shinyapps.io/citationchaser/), contacting key authors for unpublished or ongoing studies, and maintaining active surveillance of major neonatal-nutrition conferences such as those hosted by the United South African Neonatal Association (USANA), South African Paediatric Association (SAPA), Association for Dietitians in South Africa (ADSA), and the Nutrition and Growth Conference.

### Data collection and analysis

The results derived from searching the different databases will be exported into the systematic review automation tool CADIMA (CADIMA. Quedlinburg, Germany: Julius Kühn-Institut; 2017) [21]. Search results will be merged, and de-duplication will be performed, whereby, through applying the inclusion and exclusion criteria, eligible studies will be included in the review.

#### Selection of studies

Two reviewers (MS and LvW) will work independently to screen each title and abstract of studies retrieved by the literature searches, to remove any unrelated studies. Remaining studies will then be examined as full-text publications for potential inclusion. Reviewers will operate independently and in duplicate, utilising an eligibility form based on the inclusion criteria. Literature will be screened for any multiple reports of the same study. RCTs will be compared using trial identification numbers for duplicates and will allow identification and correspondence with principal investigators of ongoing trials for unpublished data (which will be included as ongoing clinical trials). Discrepancies and any disagreements will be resolved through discussion or arbitration by a third author (MvW). The selection process will be demonstrated graphically utilising a PRISMA flow diagram. Details and characteristics of excluded studies will be provided.

#### Data items extraction and management

Two review authors (MS and LvW) will independently extract study data using a standardized form entered into NMAstudio software. Any missing or ambiguous information will be clarified by contacting the original trial authors, and discrepancies between reviewers will be resolved through discussion or adjudication by a third reviewer (MvW). Extracted data will encompass bibliographic details (year, title, authorship, study design, and unit of randomization) and participant characteristics such as gestational age, birth weight, age at enrolment, and sex distribution.

Comprehensive intervention details will include the type and formulation of supplementation, comparator description, use and duration of total parenteral nutrition, timing of initiation, dosage quantification, mean duration of feeding, and attainment of full enteral feeds (defined as 120–150 mL/kg/day). Outcome data will cover both growth parameters, such as weight growth velocity (g/kg/day or Z-score change), linear length, and head-circumference velocity, and clinical outcomes, including necrotizing enterocolitis (NEC), late-onset sepsis (LOS), length of hospital stay, and mortality. All data fields and decisions will be fully documented to ensure transparency and reproducibility.

### Assessment of risk of bias in included studies

The risk of bias for each included study will be evaluated independently by two reviewers (MS and LvW). For randomized controlled trials, the revised Cochrane tool for assessing risk of bias in randomised trials (RoB 2 tool) [22] will be applied to assess potential bias across five domains: the randomization process, deviations from intended interventions, missing outcome data, measurement of outcomes, and selection of reported results.

For prospective observational studies, the ROBINS-I instrument [23, 24] will be employed to evaluate biases due to confounding, participant selection, classification of interventions, deviations, missing data, outcome measurement, and selective reporting.

Each domain will be rated as low risk, some concerns, or high risk, and an overall judgment will be summarized in tabular and graphical form. These ratings will subsequently inform sensitivity analyses and the GRADE certainty assessments for primary outcomes.

#### Summary measures of treatment effect

For dichotomous outcomes, the risk ratio (RR) with 95% confidence interval (CI) will be calculated. In the event that odds ratios are reported, using methods described in Sects. 6.4.1; and 15.4.4 of the Cochrane Handbook for Systematic Reviews of Interventions, odds ratios will be converted to risk ratios. For continuous outcomes, the mean difference (when the measured outcome is reported using the same scale) will be calculated. Alternatively, the standard mean difference (SMD) with 95% CI will be calculated. If a primary outcome (e.g., WGV) is measured both as a continuous and dichotomous outcome in the included studies, a meta-analysis will be performed and reported separately. Hazard ratio (HR) for time-to-event variables, with a 95% CI, will be calculated.

### Relative treatment ranking

Ranking probabilities will be estimated for all interventions at each possible rank. Subsequently, using the surface under the cumulative ranking curve (SUCRA), a treatment hierarchy will be established. Treatment hierarchies will be presented utilising rankograms, cumulative probability plots, and clustered ranking graphs [25]. For primary outcomes, the robustness of the findings will be assessed using a sensitivity analysis by considering estimates of mean rank with 95% CIs [26].

#### Unit of analysis issues

Adjusted estimates will be used when they are provided; if not, adjustments will be made. To prevent duplication of results, if multi-arm trials contribute multiple comparisons to a meta-analysis, treatment groups will be combined, or the shared group will be split, as appropriate.

#### Dealing with missing data

Authors of included studies will be approached for additional or missing information, if needed, to assist with the provision of additional data and study clarification. In the event that missing information cannot be acquired, it will be recorded.

When missing data is not considered to be at random, imputation methods will be employed. An attempt to calculate values from available data (e.g., SDs from standard errors) will be made. If this is not possible, imputation methods will be used [27]. Whenever possible, data will be analysed on an intention-to-treat basis. Through sensitivity analyses, the effect of imputation on the meta-analysis will be investigated.

### Assessment of heterogeneity and inconsistency

#### Assessment of clinical and methodological heterogeneity within treatment comparisons

The presence of clinical and methodological heterogeneity will be evaluated by examining the distribution of treatment effect modifiers across trials (see Subgroup analysis and investigation of heterogeneity).

#### Assessment of transitivity

The assumption of transitivity implies that the estimated effect of a particular intervention obtained through indirect comparisons is comparable to the estimated effect obtained through a direct comparison [28]. Potential effect modifiers will be investigated: gestation, sex, birth weight, small for gestational age, the presence of neonatal comorbidities, intervention type/formulation, and any co-interventions (concomitant use of parenteral nutrition, multiple nutrient supplements used simultaneously). The transitivity assumption will be evaluated by reviewing the distribution of these across the different trials (see Subgroup analysis and investigation of heterogeneity).

### Assessment of statistical heterogeneity and inconsistency

#### Pairwise comparisons

For direct treatment comparisons, Chi^2^ tests will be performed to assess statistical heterogeneity, with a significance threshold of *p* < 0.1 considered indicative of meaningful heterogeneity. The degree of inconsistency across studies will also be quantified using the I^2^ statistic, interpreted according to established thresholds: values between 0 and 40% will be regarded as likely unimportant, 30%–60% as possibly moderate, 50%–90% as potentially substantial, and 75%–100% as indicative of considerable heterogeneity.

### Geometry of the network

#### Planned method of analysis: network meta-analysis

In this protocol, the statistical manifestation of transitivity will be referred to as “inconsistency”. The presence of inconsistency will be evaluated by comparing direct and indirect treatment estimates of each treatment comparison.

To estimate the difference between direct and indirect comparisons, the node-splitting approach will be employed on all comparisons in the network [29]. If heterogeneity is identified, subgroup analyses will be conducted to identify the possible causes of heterogeneity. The importance of the identified inconsistency will be taken into account by considering the:

- choice of the question of interest (broad versus narrow questions)
- relative and absolute effects
- I.^2^ contextualization (considering small or large study effects when interpreting heterogeneity)

With the interpretation of the results obtained, consideration will be given to the above.

### Assessment of reporting biases

By means of a comparison-adjusted funnel plot, the possibility of reporting bias will be examined to highlight possible small-study effects in the NMA [26, 28].

### Data synthesis

All eligible studies will be included in the analyses; however, sensitivity analyses will be conducted according to the risk-of-bias ratings (see Sensitivity analysis). Once validity assumptions are met, an NMA will be performed. In an NMA, for every intervention, direct and indirect evidence are utilised to determine the treatment effects of each intervention. Direct evidence is obtained from direct comparisons of interventions within individual RCTs. Whereas indirect evidence is derived through combining data from multiple studies that share the same or similar comparators [30]. To incorporate both direct and indirect evidence in an NMA, the statistical model used in the analysis includes a network of evidence that links all the interventions in the network through common comparators [26].

The objectives of this NMA are predominantly to (i) ascertain the effect of all oral nutrient supplements (macro and micronutrients) currently used in VLBWIs, (ii) to evaluate the effect of each individual nutrient supplement/compound on in-hospital post-natal growth, and (iii) to examine whether the nutrient supplement/compound type, or whether the gestational age, sex and birth weight of the participants are associated with the intervention effect.

For objective (i), the different interventions will be pooled or ‘lumped’ into a single NMA comparison. Objective (ii) seeks to investigate the effect of each intervention on post-natal growth separately. This NMA will establish separate comparisons for each nutrient supplement, as well as subgrouping the ‘lumped’ comparison by macronutrient versus micronutrient used [31]. Objective (iii) will explore factors that could determine heterogeneity among the intervention effects by identifying specific characteristics as effect modifiers.

This review will include studies that compare experimental interventions with an inactive control intervention (e.g., no addition of nutrient supplement, placebo, or standard care) as well as with an active control intervention (e.g., a variant of the same intervention, a different dose of the same intervention, or different interventions). Any variations in the dose of each macro or micronutrient will be included. Comparators that are given in different doses, for different durations between the comparator groups, or in combination with other oral fortifiers or oral nutrient supplements (co-intervention) will be separated into subgroups and documented. The method of measuring, quantifying, or ensuring the exact dose of each nutrient supplement provided will also be reviewed.

Each intervention will be defined as either a macronutrient or a micronutrient oral supplement. Macronutrients will further be subdivided into (i) proteins, (ii) carbohydrates, and (iii) lipids.

Micronutrients will be subdivided into (i) macrominerals, (ii) trace elements, and (iii) vitamins, and compared to each other. The TIDieR checklist [32] will be used to identify particular characteristics that could potentially modify the effect of the interventions used. If different doses of an oral nutrient supplement are used (as opposed to placebo or no supplement), groups will be stratified into “low” or “high dose” supplementation based on the recommended ESPHGHAN nutrient guidelines [15].

### Direct comparison of interventions

Using a random-effects model, pairwise meta-analysis will be done for each direct comparison. The corresponding 95% confidence intervals (CIs) for all analyses will be calculated. Stata (StataCorp 2023; Stata Statistical Software, StataCorp LP, College Station, TX, USA) will be used for analyses, where appropriate. Subgroup analyses to explore heterogeneity will be employed should any identified trials be too clinically heterogeneous for pooling.

### Indirect comparisons of interventions

Indirect analysis will be conducted by performing subgroup analyses using standard meta-analysis routines. A p-value for the statistical significance of the difference between the subgroups based on the estimated variance of the indirect effect estimate, with the corresponding 95% CIs for the indirect summary effect, will be provided [25].

### Methods for network meta‐analysis

Data synthesis will follow a frequentist network meta-analysis framework using Stata 2023 (version 18). For each outcome, both pairwise and network-level analyses will be performed once the assumptions of homogeneity, transitivity, and consistency are met. Pairwise meta-analyses will apply random-effects models to compute risk ratios (RRs) with 95% confidence intervals for dichotomous outcomes, and mean differences (MDs) or standardized mean differences (SMDs) for continuous outcomes.

Within the network framework, relative treatment effects will be estimated through multivariate random-effects models, incorporating both direct and indirect comparisons across interventions. Node-splitting analyses will assess inconsistency between direct and indirect evidence, while χ^2^ and I^2^ statistics will quantify statistical heterogeneity. The degree of inconsistency will be further explored through subgroup and sensitivity analyses based on gestational age, birth-weight category, sex, and intervention type or formulation.

To summarize comparative effectiveness, ranking probabilities for each intervention will be derived and expressed as the surface under the cumulative ranking curve (SUCRA), supported by rankograms and cumulative probability plots. The robustness of the ranking results will be tested through sensitivity analyses that exclude studies at high risk of bias or with imputed data. All findings will be interpreted alongside GRADE assessments to ensure that effect estimates are contextualized by the certainty of the underlying evidence.

If the assumptions that preserve the validity of the NMA are not met, only direct comparisons will be made, and a narrative description of the findings will be presented.

### Subgroup analysis and investigation of heterogeneity

Subgroup analyses will be undertaken to explore potential sources of clinical and methodological heterogeneity. These analyses will examine differences across study design and setting (randomised controlled trials and prospective observational studies conducted in neonatal intensive care units or special care baby units between 2010 and 2024), as well as key participant characteristics, including sex (female versus male), gestational age categories (≤ 28 weeks, 28–32 weeks, and 32–35 weeks), and birth-weight strata (≤ 1000 g, 1000–1250 g, and 1250–1500 g).

Additional subgroup analyses will evaluate intervention-related factors, such as the nutrient type or formulation (macronutrient versus micronutrient supplementation) and the mode of feeding, distinguishing between enteral feeds administered alone and those given in combination with total parenteral nutrition. Each intervention will be summarised independently, and its effect on somatic growth and in-hospital outcomes will be compared with other nutrient interventions to assess potential differences between micro- and macronutrient supplementation strategies.

### Sensitivity analysis

Sensitivity analyses will be performed to assess the robustness and validity of the meta-analytic findings. These analyses will evaluate the impact of missing data, risk of bias, and study eligibility on the pooled estimates. To address missing data, comparisons will be made between “best-case” and “worst-case” scenarios to determine whether assumptions regarding incomplete information influence the overall results.

In addition, sensitivity analyses will be repeated after excluding studies classified as having a high risk of bias or those that only partially met the inclusion criteria, to ensure that the main findings are not disproportionately driven by lower-quality or marginally eligible trials. Any substantial change in effect estimates across these analyses will be interpreted as a potential indicator of instability in the results and discussed accordingly in the final synthesis.

### Summary of findings and assessment of the certainty of the evidence

The GRADE approach for NMAs will be used to review the certainty of the evidence [33]. For each primary outcome, the certainty of both direct and indirect evidence will be evaluated across the five standard GRADE domains: risk of bias, consistency of effects, imprecision, indirectness, and publication bias. The overall certainty of evidence for each comparison will be classified as high, moderate, low, or very low, based on the magnitude and direction of the treatment effect, the precision of the confidence intervals, and the quality of the contributing evidence.

### Thresholds for critical outcomes for imprecision judgements

Precision will be judged as adequate if the 95% confidence interval (CI) excludes a relative risk (RR) of 1.0 or if the 95% CI includes an RR of 1.0 but the CI does not include appreciable benefit or harm, and the total number of events or patients exceeds the optimal information size (OIS) [27].

Imprecision will be rated using the partially and fully contextualised GRADE approaches for making recommendations [27] rating down for imprecision by one, two, and three levels utilising GRADEPro GDT (https://www.gradepro.org/).

### Critical outcomes for decision making

Where uncertainty remains, the GRADE assessment will apply both partially and fully contextualised approaches to determine whether the effect estimates warrant rating down for imprecision by one or more levels, using the GRADEpro GDT software. For continuous outcomes, thresholds corresponding to small, moderate, or large effects will be defined to interpret the clinical importance of changes in somatic growth outcomes.

Judgements will independently be made by two review authors (MS and LvW) concerning the certainty of the evidence (high, moderate, low, or very low). Disagreements will be resolved through means of discussion or through review by a third review author (MvW). Relative effect, certainty of evidence ratings, and the absolute risks will be documented.

## Discussion

This review protocol addresses a critical evidence gap in neonatal nutrition by synthesizing the comparative effectiveness of nutrient supplementation strategies for very low birth weight infants (VLBWIs). The strength of this review lies in its use of a network meta-analysis (NMA) framework, which allows integration of both direct and indirect evidence across multiple nutritional interventions. This approach enables a comprehensive evaluation of the relative efficacy of different macro- and micronutrient supplements, something not achieved by prior pairwise meta-analyses. The inclusion of both randomized controlled trials and prospective observational studies enhances generalizability, while adherence to PRISMA-P, GRADE, and Cochrane Handbook standards ensures methodological transparency and reproducibility.

Potential limitations of this study include the expected clinical and methodological heterogeneity among studies, variations in nutrient formulations, dosing, and fortification methods, and possible inconsistency within the evidence network. The restriction to English-language publications and the last 15 years may introduce selection bias and limit the type of nutrient studies included, though this time frame aligns with the evolution of neonatal nutrition practices. Small sample sizes in neonatal trials and incomplete outcome reporting may limit the precision of pooled estimates. To mitigate these challenges, we will apply robust sensitivity analyses, node-splitting methods to test for inconsistency, and GRADE certainty assessments.

The findings of this review will have important implications for clinical practice and research. By ranking nutrient supplementation strategies based on their comparative effectiveness, this study will inform standardized feeding protocols and guide clinical decision-making for optimizing early growth in VLBWIs. Furthermore, it will identify key research gaps, such as unexplored combinations of macro- and micronutrients, that can guide future neonatal nutrition clinical trials.

## Conclusion

This NMA will provide evidence-based guidance on optimal nutrient supplementation strategies to enhance postnatal growth in VLBWIs, addressing a critical knowledge gap in neonatal nutrition practices.

## Supplementary Information

Supplementary Material 1.

## Data Availability

As this is a protocol for a systematic review and network meta-analysis, no primary data were generated. Upon completion of the review, all data extracted and analyzed will be made available in a publishable format, as supplementary material or upon reasonable request to the corresponding author.

## References

[1] Riddle WR, DonLevy SC, Qi XF, Giuse DA, Rosenbloom ST. Equations to support predictive automated postnatal growth curves for premature infants. J Perinatol. 2006;26(6):354–8. 16688206 10.1038/sj.jp.7211511

[2] Ehrenkranz RA. Early aggressive nutritional management for very low birth weight infants: what is the evidence? Semin Perinatol. 2007;31(2):48–55. 17462488 10.1053/j.semperi.2007.02.001

[3] Goldberg DL, Becker PJ, Brigham K, Carlson S, Fleck L, Gollins L, et al. Identifying malnutrition in preterm and neonatal populations: recommended indicators. J Acad Nutr Diet. 2018;118(9):1574–82. 10.1016/j.jand.2017.10.006 29398569

[4] Clouchoux C, Guizard N, Evans AC, Du Plessis AJ, Limperopoulos C. Normative fetal brain growth by quantitative in vivo magnetic resonance imaging. Am J Obstet Gynecol. 2012;206(2):173.e1-173.e8. 22055336 10.1016/j.ajog.2011.10.002 PMC13054723

[5] Kleinman RE. Pediatric Nutrition. 8th ed. American Academy of Pediatrics; 2019. Available from: https://www.perlego.com/book/1320877/pediatric-nutrition-pdf. Cited 2023 Aug 15.

[6] Peila C, Spada E, Giuliani F, Maiocco G, Raia M, Cresi F, et al. Extrauterine Growth Restriction: Definitions and Predictability of Outcomes in a Cohort of Very Low Birth Weight Infants or Preterm Neonates. 2020 May 1;12(5). Available from: https://pubmed.ncbi.nlm.nih.gov/32357530/. 10.3390/nu12051224 PMC7281990 32357530

[7] Lan S, Fu H, Zhang R, Zhong G, Pan L, Bei F, et al. Extrauterine growth restriction in preterm infants: Postnatal growth pattern and physical development outcomes at age 3–6 years. Front Pediatr. 2022;29(10):1248. 10.3389/fped.2022.945422 PMC9372328 35967552

[8] Arslanoglu S, Moro GE, Ziegler EE. Adjustable fortification of human milk fed to preterm infants: does it make a difference? J Perinatol. 2006;26(10):614–21. 16885989 10.1038/sj.jp.7211571

[9] Alan S, Atasay B, Cakir U, Yildiz D, Kilic A, Kahvecioglu D, et al. An intention to achieve better postnatal in-hospital-growth for preterm infants: Adjustable protein fortification of human milk. Early Hum Dev. 2013;89(12):1017–23. 24035039 10.1016/j.earlhumdev.2013.08.015

[10] Brown JV, Embleton ND, Harding JE, Mcguire W. Multi-nutrient fortification of human milk for preterm infants. Cochrane Database Syst Rev. 2016;2016(5):CD000343. 10.1002/14651858.CD000343.pub3 27155888

[11] Brown JVE, Lin L, Embleton ND, Harding JE, McGuire W. Multi‐nutrient fortification of human milk for preterm infants. 2020 June 3;2020(6). Available from: /pmc/articles/PMC7268980/10.1002/14651858.CD000343.pub4 PMC7268980 35658821

[12] Harding JE, Wilson J, Brown J. Calcium and phosphorus supplementation of human milk for preterm infants. Cochrane Database Syst Rev. 2017;2(2):CD003310. 28238222 10.1002/14651858.CD003310.pub2 PMC6464224

[13] Arslanoglu S, Boquien CY, King C, Lamireau D, Tonetto P, Barnett D, et al. Fortification of human milk for preterm infants: update and recommendations of the European milk bank association (EMBA) working group on human milk fortification. Front Pediatr. 2019;7:76. 30968003 10.3389/fped.2019.00076 PMC6439523

[14] Chitale R, Ferguson K, Talej M, Yang WC, He S, Edmond KM, et al. Early enteral feeding for preterm or low birth weight infants: a systematic review and meta-analysis. Pediatrics. 2022;150(Suppl 1):e2022057092E. 35921673 10.1542/peds.2022-057092E

[15] Embleton ND, Jennifer Moltu S, Lapillonne A, van den Akker CHP, Carnielli V, Fusch C, et al. Enteral Nutrition in Preterm Infants (2022): A Position Paper From the ESPGHAN Committee on Nutrition and Invited Experts. J Pediatr Gastroenterol Nutr. 2023;76(2):248–68. 36705703 10.1097/MPG.0000000000003642

[16] Amissah EA, Brown J, Harding JE. Fat supplementation of human milk for promoting growth in preterm infants - Amissah, EA - 2020 | Cochrane Library. 2020; Available from: https://www.cochranelibrary.com/cdsr/doi/10.1002/14651858.CD000341.pub3/full. Cited 2024 Oct 28. 10.1002/14651858.CD000341.pub3 PMC8236752 32842164

[17] Amissah EA, Brown J, Harding JE. Protein supplementation of human milk for promoting growth in preterm infants. Cochrane Database Syst Rev. 2020;2020(9):000433. 10.1002/14651858.CD000433.pub3 PMC8094919 32964431

[18] Tao J, Yang J, Li Y, Su Y, Mao J. Effectiveness of feeding supplementation in preterm infants: an overview of systematic reviews. 2020. 10.1186/s12887-021-03052-w PMC8725413 34983444

[19] Kumar M, Chowdhury R, Sinha B, Upadhyay RP, Chandola TR, Mazumder S, et al. Enteral multiple micronutrient supplementation in preterm and low birth weight infants: a systematic review and meta-analysis. Pediatrics. 2022;150(Supplement 1):e2022057092N. 35921670 10.1542/peds.2022-057092N

[20] Agostoni C, Buonocore G, Carnielli VP, De Curtis M, Darmaun D, Decsi T, et al. Enteral nutrient supply for preterm infants: commentary from the European Society of Paediatric Gastroenterology, Hepatology and Nutrition Committee on Nutrition. J Pediatr Gastroenterol Nutr. 2010;50(1):85–91. 19881390 10.1097/MPG.0b013e3181adaee0

[21] Kohl C, Mcintosh E, Unger S, Haddaway N, Kecke S, Schiemann J, et al. Online tools supporting the conduct and reporting of systematic reviews and systematic maps: A case study on CADIMA and review of existing tools. Environ Evid. 2018;7:8.

[22] Sterne JAC, Savović J, Page MJ, Elbers RG, Blencowe NS, Boutron I, et al. RoB 2: a revised tool for assessing risk of bias in randomised trials. BMJ. 2019;28(366):l4898. 10.1136/bmj.l4898 31462531

[23] Sterne JA, Hernán MA, Reeves BC, Savović J, Berkman ND, Viswanathan M, et al. ROBINS-I: a tool for assessing risk of bias in non-randomised studies of interventions. 2016 Oct 12; Available from: https://www.bmj.com/content/355/bmj.i4919. Cited 2025 Nov 18. 10.1136/bmj.i4919 PMC5062054 27733354

[24] Igelström E, Campbell M, Craig P, Katikireddi SV. Cochrane’s risk of bias tool for non-randomized studies (ROBINS-I) is frequently misapplied: A methodological systematic review. J Clin Epidemiol. 2021;1(140):22–32. 10.1016/j.jclinepi.2021.08.022 PMC8809341 34437948

[25] Chaimani A, Higgins JPT, Mavridis D, Spyridonos P, Salanti G. Graphical tools for network meta-analysis in STATA. PLoS ONE. 2013;8(10):e76654. 24098547 10.1371/journal.pone.0076654 PMC3789683

[26] Salanti G, Ades AE, Ioannidis JPA. Graphical methods and numerical summaries for presenting results from multiple-treatment meta-analysis: an overview and tutorial. J Clin Epidemiol. 2011;64(2):163–71. 20688472 10.1016/j.jclinepi.2010.03.016

[27] Higgins JP, Li T, Deeks JJ. Chapter 6: Choosing effect measures and computing estimates of effect. 2023.

[28] Chaimani A, Caldwell DM, Li T, Higgins JPT, Salanti G. Additional considerations are required when preparing a protocol for a systematic review with multiple interventions. J Clin Epidemiol. 2017;1(83):65–74. 10.1016/j.jclinepi.2016.11.015 28088593

[29] Dias S, Welton NJ, Caldwell DM, Ades AE. Checking consistency in mixed treatment comparison meta-analysis. Stat Med. 2010;29(7–8):932–44. 20213715 10.1002/sim.3767

[30] Vilyte G, Fox T, Rohwer AC, Volmink J, McCaul M. Digital devices for tuberculosis treatment adherence. 2023;(6). Available from: 10.1002/14651858.CD015709/full

[31] Thomas J, Kneale D, McKenzie JE, Brennan SE, Bhaumik S. Chapter 2: Determining the scope of the review and the questions it will address. 2023. Available from: https://www.cochranelibrary.com/cdsr/doi/10.1002/14651858.CD015709/full.

[32] Hoffmann TC, Glasziou PP, Boutron I, Milne R, Perera R, Moher D, et al. Better reporting of interventions: template for intervention description and replication (TIDieR) checklist and guide. BMJ. 2014;7(348):g1687. 10.1136/bmj.g1687 24609605

[33] Puhan MA, Schünemann HJ, Murad MH, Li T, Brignardello-Petersen R, Singh JA, et al. A GRADE working group approach for rating the quality of treatment effect estimates from network meta-analysis. BMJ. 2014;349(24):g5630. 25252733 10.1136/bmj.g5630

