# Supplementary material for: Effect of nutrient supplementation on somatic growth in very low birth weight infants: a protocol for a systematic review and network meta-analysis
Source: Syst Rev. 2026 Feb 19;15:98. doi: 10.1186/s13643-025-03027-3 (PMC13020165; doi:10.1186/s13643-025-03027-3)
Supplement: Supplementary file 1 — Supplementary Material 1. [file 13643_2025_3027_MOESM1_ESM.docx]

APPENDICES

Appendix 1. Study definitions

Note: Definitions adapted from Vermont Oxford Network (Available at: https://vtoxford.zendesk.com/hc/en-us/articles/4405064008467-2022-Manual-of-Operations-Part-2-Release-26-2-PDF- )

- Very low birth weight infants: All neonates born with a birth weight less than 1500g.
- Extremely low birth weight: All neonates born with a birth weight less than 1000 g
- Small for gestational age defined as weight <10^th^ centile according to Intergrowth 21^st^ standards, based on the birth weight, gestation, and gender.
- Day of First feed: First day where at least 6 of the first prescribed feeds are given.
- Full feeds: All feeds received via nasogastric/orogastric tube or orally at a volume of 150ml/kg/day.
- Fortification: addition of a single or a combination of micro and macronutrients to feeds
- Supplement: addition of minerals, trace elements, or vitamins to the diet
- Growth velocity: calculated using a 2-point model, using net weight gain over the time interval divided by the time interval and average weight (g/kg/day)
- Necrotising enterocolitis classified according to the Modified Bell’s Staging criteria. Must be diagnosed at surgery, at post-mortem examination, or with clinical and diagnostic imaging using the following criteria

- At least one of the following clinical signs present:

-Bilious gastric aspirate or emesis

- Abdominal distension or discoloration

- Occult or gross blood in stool (no fissure), and at least one of the following diagnostic imaging findings present:

-Pneumatosis intestinalis

- Hepato-biliary gas

- Pneumoperitoneum

- Sepsis: Blood stream infection associated with a raised CRP (>10 mg/l) or growth of a bacteria on blood culture.
- Early onset neonatal sepsis: defined as sepsis in a neonate in the first 72 hours of life.
- Late onset sepsis: defined as sepsis in a neonate after the first 72 hours of life.

Appendix 2. PubMed search strategy

Premature Birth[Mesh Terms] OR Infant, Very Low Birth Weight[Mesh Terms] OR Infant, Extremely Low Birth Weight[Mesh Terms] OR Infant, Newborn[Mesh Terms] OR neonate[Mesh Terms] OR (“Extremely Premature Infant” OR “Premature Births” OR “Preterm Birth” OR “Premature Infant” OR “Neonatal Prematurity” OR “Preterm Infant” OR “Very-Low-Birth-Weight Infant” OR “Very Low Birth Weight Infant” OR “Very Low Birth Weight” OR “Extremely Low Birth Weight Infant”

**AND**

Infant Nutritional Physiological Phenomena[Mesh Terms] OR Breast Feeding[Mesh Terms] OR Bottle Feeding[Mesh Terms] OR Enteral Nutrition[Mesh Terms] OR Nutrients[Mesh Terms] OR Milk, Human[Mesh Terms] OR Infant Formula[Mesh Terms] OR Dietary Supplements[Mesh Terms] OR Food, Fortified[Mesh Terms] OR (“Infant Nutritional Physiological Phenomen*” OR “Infant Nutrition Physiology” OR “Infant Nutritional Physiology” OR “Supplement* Feed*” OR “Complement* Feed*” OR “Bottle Feed*” OR “Breast Feed*” OR “Breast Milk Expression” OR Wean* OR “human milk” OR “breast milk” OR “infant formula” OR “baby formula” OR micronutrient* OR macronutrient* OR vitamin* OR mineral* OR “medium chain triglyceride” OR omega-3 OR omega-6 OR arginine OR calcium OR copper OR “folic acid” OR glutamine OR iodine OR iron OR phosphorus OR phosphate OR selenium OR sodium OR “supplement* food” OR “fortifi* food” OR “enriched food” OR nutri* OR multinutrient* OR multi-nutri* OR “enteral feed*” OR “enteral nutri*” OR “diet* supplement*” OR “food supplement*” OR “oral feed” OR “tube feed*” OR “Gastric Feeding Tubes” OR “Force Feed*” OR “milk protein*” OR “diet* protein*” OR “diet* carb*” OR “diet* fat*”)

**AND**

Intensive Care Units[Mesh Terms] OR Intensive Care Units, Neonatal[Mesh Terms] (“paediatric* hospital*” OR Hospital* OR “pediatric* hospital*” OR “newborn hospital*” OR “neonat* hospital*” OR “intensive care*” OR ICU OR NICU OR “paediatric* unit” OR “pediatric* unit” OR “newborn unit” OR “neonat* unit” OR in-hospital )

**AND**

("randomized controlled trial"[Publication Type] OR "controlled clinical trial"[Publication Type] OR "randomized"[Title/Abstract] OR "placebo"[Title/Abstract] OR "drug therapy"[MeSH Subheading] OR "randomly"[Title/Abstract] OR "trial"[Title/Abstract] OR "groups"[Title/Abstract]) NOT ("animals"[MeSH Terms] NOT "humans"[MeSH Terms])

**OR**

“Epidemiologic Studies”[MeSH] OR “case control”[TIAB] OR “case-control”[TIAB] OR ((case[TIAB] OR cases[TIAB]) AND (control[TIAB] OR controls[TIAB)) OR “cohort study”[TIAB] OR “cohort analysis”[TIAB] OR “follow up study”[TIAB] OR “follow-up study”[TIAB] OR “observational study”[TIAB] OR longitudinal[TIAB] OR retrospective[TIAB] OR “cross sectional”[TIAB] OR questionnaire[TIAB] OR questionnaires[TIAB] OR survey[TIAB

**PubMed**

| # | Search | Results |
| --- | --- | --- |
| #13 | #11 AND #12 | 1400 |
| #12 | ("2010/01/01"[Date - Publication] : "3000"[Date - Publication]) | 17 105 420 |
| #11 | #3 AND #6 AND #9 AND #10 | 1 958 |
| #10 | (randomized controlled trial[Publication Type] OR (randomized[Title/Abstract] AND controlled[Title/Abstract] AND trial[Title/Abstract])) | 688 577 |
| #9 | #7 OR #8 | 7 195 965 |
| #8 | Hospital[Title/Abstract] OR "pediatric hospital"[Title/Abstract] OR "neonatal hospital"[Title/Abstract] OR "neonatal intensive care unit"[Title/Abstract] OR "neonatal ICU"[Title/Abstract] OR "neonatal unit"[Title/Abstract] OR "pediatric unit"[Title/Abstract] | 1 333 928 |
| #7 | Hospital OR hospital. pediatric[MeSH Terms] | 7 189 215 |
| #6 | #4 OR #5 | 4 406 149 |
| #5 | "Dietary Supplement"[Title/Abstract] OR "Fortified Food"[Title/Abstract] OR "Supplemented Food"[Title/Abstract] OR "Breast Milk Expression"[Title/Abstract] OR protein[Title/Abstract] OR carbohydrates[Title/Abstract] OR "dietary fats"[Title/Abstract] OR "medium chain triglycerides"[Title/Abstract] OR "linolenic acid"[Title/Abstract] OR "linoleic acid"[Title/Abstract] OR "olive oil"[Title/Abstract] OR "soybean oil"[Title/Abstract] OR zinc[Title/Abstract] OR "vitamin A"[Title/Abstract] OR "vitamin D"[Title/Abstract] OR "vitamin E"[Title/Abstract] OR "vitamin C"[Title/Abstract] OR sodium[Title/Abstract] OR iron[Title/Abstract] OR arginine[Title/Abstract] OR glutamine[Title/Abstract] OR selenium[Title/Abstract] OR phosphate[Title/Abstract] OR copper[Title/Abstract] OR "folic acid"[Title/Abstract] | 4 275 311 |
| #4 | Dietary Supplements OR Food, Fortified OR Breast Milk Expression OR Dietary Fats[MeSH Terms] | 223 833 |
| #3 | #1 OR #2 | 1 032 471 |
| #2 | Preterm[Title/Abstract] OR Premature[Title/Abstract] OR prem[Title/Abstract] OR "Extremely Premature Infant"[Title/Abstract] OR "Premature Births"[Title/Abstract] OR "Preterm Birth"[Title/Abstract] OR "Premature Infant"[Title/Abstract] OR "Neonatal Prematurity"[Title/Abstract] OR "Preterm Infant"[Title/Abstract] OR "Very-Low-Birth-Weight Infant"[Title/Abstract] OR "Very Low Birth Weight Infant"[Title/Abstract] OR "Very Low Birth Weight"[Title/Abstract] OR infant[Title/Abstract] OR "Extremely Low Birth Weight Infant"[Title/Abstract] OR "newborn infant"[Title/Abstract] OR newborn[Title/Abstract] OR neonate[Title/Abstract] | 593 061 |
| #1 | Infant, extremely premature OR Premature birth OR Infant, Premature OR Infant, Extremely Low Birth Weight OR Infant, Very Low Birth Weight OR Infant, Newborn[MeSH Terms] | 708 868 |

Appendix 3. Fortifiers and nutrient supplements to improve growth

| Nutrient supplement (15,19,78) | Principle | Purpose | Growth Advantages |
| --- | --- | --- | --- |
| **Single nutrient fortifiers** | - Addition of single macronutrients to feeds - Useful for individualizing fortification | | |
| Protein | - Partially hydrolysed protein source - Protein supplementation to reach intake from 3.5 - 4.5 g/kg/d | - protein intake is the main driver of lean body mass growth provided there’s sufficient energy intake | - Several RCTs comparing higher versus moderate protein intakes - Supports somatic growth (including head growth) |
| Carbohydrates | - Dextrin maltose, lactose, glucose, galactose, HMOs | - Carbohydrates constitute 45%–50% of nonprotein calories in EBM | - observational studies conclude that increasing energy with carbohydrate together with protein regimens is safe and improve in-hospital growth |
| Lipids | - Linoleic, linolenic acids, medium chain triglycerides, DHA, olive oil - studies show a decrease in ARA and DHA levels in preterm infants after birth | - Detary fats provide about 50% of the energy needs of preterm infants as well as essential polyunsaturated fatty acids (PUFAs), lipid-soluble vitamins, and complex lipids | - Data from meta-analysis and RCTs on the effect of supplementation on neurodevelopmental and growth show inconsistent results |
| **Multi-nutrient fortifiers** | - Addition of fortifier that contains a combination of macro/micronutrients, minerals, trace elements and vitamins   Reach ESPGHAN recommendations for nutrients to improve post-natal growth   - Increases in‐hospital rate of weight gain, body length and head circumference among preterm infants (55) | | |
| **Macro-minerals** | - Addition of single minerals to feeds or oral supplementation | | |
| Sodium | - Concentration of sodium in EBM declines rapidly over the first few postnatal days - Addition of 5% sodium to feeds | - Has a role in bone mineralization, nerve conduction, nitrogen retention, and growth | - High-quality RCTs show that Na supplementation with intakes of 4–6 mmol/kg/d versus 3–4 mmol/kg/d increase weight gain |
| Chloride | - Daily turnover is high - Renal tubular reabsorption rate is 60%–70% | - Low intakes can lead to failure to thrive, slower growth, and delayed neurological development | - Improves growth and neurological development |
| Potassium | - Needed for somatic growth - Levels correlates well with lean body mass | - Crucial for maintaining muscle contractility and neuronal function | - Linear association between potassium needs and protein retention in - enterally fed preterm infants - Intake important in growing infants receiving the upper ranges of energy and protein intakes |
| Calcium | - Bone mineral metabolism |  | - Minimal data on effect on growth - Reduction in fractures, osteopenic changes on X-ray |
| Magnesium | - Bone mineral metabolism and calcium, phosphate homeostasis |  | - No RCTs determining effects on bone accretion and growth |
| Phosphate | - Bone mineral metabolism - Involved in lean mass accretion - Used in the intra-cellular energy metabolism |  | - Minimal data on effect on growth - Reduction in fractures, osteopenic changes on X-ray |
| **Trace elements** | - Essential for different organ system functions - Needed for normal growth and development | | |
| Iron | - Maintenance of haemoglobin levels | - Supplements effectively prevent iron deficiency anaemia and its impact on growth | - Improved developmental outcomes |
| Zinc | - Involved in growth and tissue differentiation | - Deficiency associated with poor growth, increased risk for infections, skin rash, poor neurodevelopment | - Improves weight gain and linear growth |
| Copper | - Essential nutrient with multiple functions as part of enzymes | - Low birth weight risk factor for copper deficiency | - Very few clinical trials of different copper intakes and effect on growth |
| Selenium | - Concentrations in breast milk are associated with maternal selenium intake - Plasma concentrations decrease during the first weeks of life in preterm infants | - Plays important role as a component of antioxidant enzymes and deiodinases required for the metabolism of thyroid hormones - Prems at high risk for oxidative stress related disorders (bronchopulmonary dysplasia, retinopathy of prematurity and cerebral white matter injury | - Data shows minimal to no effect on growth |
| Manganese | - Cofactor for many enzymes | - Important for cellular defence against free oxygen radicals | - No published data on growth benefits |
| Iodine | - Integral to thyroid hormones production | - Deficiency related to hypothyroidism, goitre, mental retardation, poor growth - Increased neonatal and infant mortality | - Currently no convincing evidence of beneficial clinical effects of iodine supplementation on growth |
| Chromium | - Essential nutrient that potentiates the action of insulin | - Improves glucose tolerance | - Insufficient data |
| Molybdenum | - Essential cofactor for several enzymes | - Involved in oxidation and reduction processes | - Insufficient data |
| **Vitamins**  **Water Soluble Vitamins** | - Essential for whole body function and homeostasis | | |
| Thiamine | - Concentration higher in term compared to preterm human milk - Decline blood thiamine concentrations with postnatal age | - Co-enzyme needed for energy metabolism - Low weight at birth related to low vitamin concentrations | - No RCTs related to effect on growth |
| Pantothenic acid | - Essential for fatty acid synthesis - Needed for oxidative degradation of fatty acids and amino acids | - Prematurity related to low concentrations in breast milk | - No RCTs related to effect on growth |
| Biotin | - Critical role in synthesis of fatty acids and gluconeogenesis | - Improves glucose tolerance | - Insufficient data |
| Niacin | - Involved in oxidation/reduction reactions | - Aassociated with catabolic and anabolic processes | - No RCTs related to effect on growth |
| Vitamin C | - Is an enzyme cofactor in biochemical reactions | - Important role in biosynthesis of collagen - Involved in metabolism of cholesterol to bile acids | - No RCTs related to effect on growth |
| Riboflavin | - Cofactors of a variety of flavoprotein enzymes | - Essential role in redox reactions foe energy metabolism, metabolic pathways and formation of some vitamins and coenzymes | - Insufficient data |
| Pyridoxine | - Cofactors of enzymes | - Involved in amino acid metabolism, glycogenolysis, gluconeogenesis, haem synthesis, lipid metabolism | - No RCTs related to effect on growth |
| Folate | - Cofactors for enzymes | - Necessary for the synthesis of RNA and DNA | - Intake associated with increased weight and length gain |
| Cobalamin | - Coenzyme | - Necessary for metabolic processes in mitochondria | - No RCTs related to effect on growth |
| **Fat Soluble Vitamins** |  | | |
| Vitamin K | - Lipophilic, hydrophobic vitamins - Necessary for synthesis of coagulation factors (factors II (prothrombin), VII, IX, and X, and anticoagulation proteins C and S in the liver | - Very low maternal transfer across the placenta - Low infant levels associated with vitamin K deficiency bleeding | - There are no RCTs in preterm infants - Nutritional recommendations vary |
| Vitamin E | - Antioxidant activity | - Low concentrations were found at birth and at discharge in preterm infants - Serum concentrations may not reflect tissue concentrations | - No clinical benefits have been seen |
| Vitamin D | - Critical role in bone metabolism and function of innate immune system | - Crucial for optimal calcium absorption | - Few RCTs showing benefit for bone mineralisation and growth |
| Vitamin A | - Essential micronutrient for growth and tissue differentiation | - Preterm infants have lower plasma concentrations of retinol and retinol binding protein at birth compared with term counterparts (reflecting low hepatic stores) | - No RCTs related to effect on growth |
